# Internet-delivered treatment: its potential as a low-intensity community intervention for adults with symptoms of depression: protocol for a randomized controlled trial

**DOI:** 10.1186/1471-244X-14-147

**Published:** 2014-05-21

**Authors:** Derek Richards, Ladislav Timulak, Gavin Doherty, John Sharry, Amy Colla, Ciara Joyce, Claire Hayes

**Affiliations:** 1 SilverCloud Health Ltd, Dublin, Ireland; 2 School of Psychology, Trintiy College, Dublin, Ireland; 3 School of Psychology, University of Dublin, Trintiy College, Dublin, Ireland; 4 School of Computer Science and Statistics, University of Dublin, Trintiy College, Dublin, Ireland; 5 Mater Misericordiae University Hospital, Dublin, Ireland; 6 Aware Charity Ireland, 72 Lower Leeson Street, Dublin 2, Ireland

**Keywords:** Depression, Online interventions, Treatment, CBT, Randomized trial, Symptoms, Internet-delivered

## Abstract

**Background:**

Depression is a high prevalence disorder, displaying high rates of lifetime incidence, early age onset, high chronicity, and role impairment. In Ireland 12-month prevalence of depression has been reported to be 10.3%. A large percentage of affected individuals have no medical diagnosis nor seek treatment. Cognitive Behavior Therapy (CBT) has established itself as an option for the treatment of depression. Many Irish adults with depression find it difficult to access evidence-based CBT, this is due to several factors, like stigma and costs. However, systematic factors including the shortage of trained professionals and the relative underdevelopment of services also make access difficult.

Stepped-care can increase access to evidence-based CBT. One option is tailored internet-delivered treatment programs. Preliminary research from Ireland needs now to include large-scale studies on effectiveness. Thus the current study seeks to examine the potential of an internet-delivered low-intensity treatment for symptoms of depression in an Irish adult community sample.

**Method/Design:**

The study is a randomized controlled trial of an online CBT (iCBT) program for the treatment of adults with depressive symptoms. The trial will include an active treatment group and a waiting-list control group. The active condition will consist of 8 weekly modules of iCBT, with post-session feedback support. Participants in the waiting list will receive access to the treatment at week 8. Participants will complete the Beck Depression Inventory (BDI-II) and eligibility criteria will also apply. Primary outcomes are depressive symptoms. Secondary outcomes include quality of life indicators, significant events and satisfaction with online treatment. Data will be collected at baseline and at post-treatment, week 8, and at follow-up week 20 (3-months) and week 32 (6-months). Analysis will be conducted on the intention-to-treat basis.

**Discussion:**

The study seeks to evaluate the effectiveness of an online delivered treatment for depression in a community sample of Irish adults with symptoms of depression. The study will be a first contribution and depending on the sample recruited the results may be generalizable to people with similar difficulties in Ireland and may therefore give insight into the potential of low-intensity interventions for Irish people with depressive symptoms.

**Trial registration number:**

Current Controlled Trials ISRCTN03704676. DOI: 10.1186/ISRCTN03704676

## Background

Depression has been ranked high among the leading causes of disease burden throughout the world [1], displaying high rates of lifetime incidence, early age onset, high chronicity, and role impairment [2]. Twelve-month prevalence rates for depression in the U.S. have been estimated at 6.6% [3], and in Europe 8.5% [4]. In Ireland 12-month prevalence of depression as measured against DSM-IV criteria has been reported to be 10.3% [4].

Studies have reported higher rates for women than for men, in Europe 12-month prevalence has been reported at 10.5% for women and 6.61% for men [4]. Another European study that examined prevalence for older adults (65+) reported an overall 12-month prevalence rate of 12.3% [5]. The study highlighted the traditional gender divide with women (14.1%) reporting a higher prevalence of depression than men (8.6%) [5].

Epidemological studies have highlighted the occurrence of depression in younger age groups. A review of the literature posits the peak years for onset to be between 15–29 years [6]. Prevalence rates and gender differences are relatively constant across the adult lifespan and given the earlier age onset would suggest that lifetime prevalence will be higher in the future for younger cohorts [6].

Depression exacts significant economic, personal, intra-personal and societal costs. The economic costs alone are justification for accurately understanding and treating depression in individuals [2,7,8]. Depressive disorders are associated with losses in quality of life and increased mortality rates [9,10].

### Treating depression

The aetiology of depression is dependent on the individual and their biological and psychosocial predisposition. The relative importance of these factors vary across individuals. The current biologic understanding of depression posits that many aspects of depression can be understood in terms of dysregulation of the Central Nervous System (CNS) responses to stress and naturally dysregulation is influenced by a wide range of moderating variables such as genetics, age, sex, and developmental history. Science has been successful in identifying key neurobiological processes and developing medications to compensate for dysregulation in the CNS.

Depression can be treated successfully using antidepressants, but relapse is high following cessation, and many patients prefer psychological therapies [11], which have proved equally as effective as antidepressants [12]. By far the most extensively researched is cognitive-behavior therapy (CBT) [13]. CBT has established itself as an option for the treatment of depression, for post-treatment gains and also for maintaining gains and preventing future relapses and recurrences [14].

CBT is an amalgmation of cognitive science and behavioral science. A central construct is that of cognitive vulnerability, where certain beliefs constitute a vulnerability to depression [15]. Such vulnerability has evolved out of peoples historical experience, the consequent core beliefs they hold about themselves, others and the world, and their information processing capabilities that are generally negatively attuned; each of which interact with the other to produce a cognitive vulnerabiltiy in the person [16]. Research has provided compelling support for cognitive-vulnerability as theorized by Beck [17] and demonstrates that cognitive-vulnerability increases the risk for first episode and recurrences of depression [18].

Vulnerability is viewed as dormant until activated by some experience that has the effect of disrupting a person’s stability or physiological and psychological homeostasis. The cause can be either one major event or experience, or an accumulation of smaller experiences [19]. Collectively they are grouped under the rubric of stress. The majority of research investigating possible links in depression consistently find a link between the experience of a stressful life event and the onset of depression [20].

Cognitive-behavioral treatment consists of a range of cognitive and behavioral strategies that aims to correct distressing beliefs and thinking that occurs in information processing with depression prone individuals, in doing so distress is alleviated and strategies and skills are taught to help the person cope more effectively with life’s challenges [16]. Treatment therefore includes self-monitoring and thought recording, behavioural activation, cognitive restructuring, challenging core beliefs, among others.

### Access to treatment

Worldwide, a large percentage of affected individuals have no medical diagnosis nor seek treatment [21]. One study estimated the worldwide treatment gap in depression at 56.3% [22]. Several barriers to accessing treatment exist, such as waiting lists, lack of motivation for change, negative perception of psychological and/or drug treatments, costs, and personal difficulty such as stigma; each can play an important role in choosing to seek diagnosis and treatment [22,23]. Of those that may seek the help they need they are often met with a shortage of trained professionals and consequent waiting lists, which contradicts the benefits of early intervention [24].

Many in Ireland with depression find it difficult to access appropriate and evidence-based CBT, this is due to several factors, some of which were mentioned earlier as barriers, but particularly in Irelands case, the shortage of trained professionals alongside the relative underdevelopment of services makes it difficult to access treatments [25]. Many Irish present their psychological difficulties, initially, to their primary care phsician however, one survey reported that less than one third of GPs have postgraduate training in psychological therapies and 85% referred less than 5% of their service users with mental health difficulties to specialists [26]. More recent reports also corroborate the state of affairs where less than 20% of patients presenting with mental health difficulties at Irish general practitioners are in receipt of secondary specialist services [27,28].

### Stepped care models of treatment

In recent years attempts to overcome barriers to access have been addressed through the evolution of a new understanding in mental health care that recognizes high-intensity (e.g. face-to-face therapy) and low-intensity (e.g. bibliotherapy) services. Low-intensity interventions signify treatments that limit specialist therapist time, or use this time in a cost effective manner, for example, group treatments [29]. To increase access to evidence-based psychological therapies it will be necessary to develop and deploy a stepped-care model where low-intensity interventions are provided as a first option, before referral to high-intensity interventions. Such a program is recommended as best-practice by the National Institute for Health and Clinical Excellence [13] and also advocated by the Irish health service [30]. Yet, despite the barriers that exist for Irish people, both personal and systemic, what is considered best-practice has not evolved in Ireland. Given the continued growth in high prevalence disorders (depression and anxiety) there is a real need to increase access. The experience of our neighbours in the UK through the Improving Access to Psychological Therapies program that uses a stepped-care model, introducing both low- and high-intensity interventions, has proven successful [31].

### Delivering low-intensity interventions for depression

One of a range of low-intensity interventions is the development and delivery of tailored treatment programs for specific disorders using the internet, as both clinican-guided and self-administered interventions [32]. Indeed CBT has proved a suitable evidence-based option for integration as a low-intensity intervention within a stepped care model [33]. More precisely, low-intensity internet-delivered treatments for depression have established a sound base supporting their effectiveness and efficacy [34]. In addition, such studies for depression treatment that provide human support yield enhanced results compared to those with no support [34,35].

The internet offers the possibility of delivering a treatment intervention at low cost and perhaps overcoming some of the barriers to access mentioned earlier. The internet can deliver treatment incrementally and in an engaging way. Furthermore, internet penetration in Ireland is at 76.8% [36] and it is likely that it is an attractive medium as people are already high users of the internet and related tools.

A number of small scale studies of online delivered treatments have been experimented with in Ireland [37,38]. There is now a need to develop larger community based projects like those that have occurred throughout Europe to assess the feasibility, efficacy effectiveness and scalability of such low-intensity interventions for Ireland. Because high-intensity interventions cannot be delivered en-mass, Ireland, like its European neighbours, will have to consider the implementation of stepped-care that will include a broad range of low-intensity interventions as first options in care.

Therefore using established CBT principles informing skills and strategies for the management of depression in an integrated disorder-specific treatment plan, the study aims to deploy these using a novel computer-delivered software platform (SilverCloud) to help demonstrate its feasibility, efficacy and effectiveness as a low-intensity intervention for depression treatment. The platform being used integrates a number of innovative engagement strategies for improving the user experience: personal, interactive, supportive and social [38]. The details of the platform and the content of treatment are described in more detail below.

### Dropout from online treatments

Although several online studies have reported benefits for users who have not completed the entire course of treatment [39] it is still the case that dropout is a continued cause of concern, especially for self-administered treatments that do not involve a therapist or other form of support to users [40]. Information to date on reasons for dropout is limited. Some studies have report variables such as difficulties using the computer, negative features of the program, perceived as too demanding, poor clinical progress, receiving alternative treatment, feeling better, lack of time, and problems understanding the computer program [34,41]. These are useful and more indept exploration of these and other potential reasons is needed so as to inform both the development and adequate implementation of online delivered treatments. The SilverCloud platform that is been used in the current study has been designed by a multi-disciplinary team of computer scientests, clinical personnel, and academics. It therefore includes a number of engagement strategies for improving the user experience (personal, interactive, supportive and social) and supporting retention.

### Aware

Aware is a charity that aims to create a society where those with depression or related mood disorders, and their families, are understood and supported, are free from stigma and have access to a broad range of support options. The charity has existed since 1985 and their objectives are, first, to provide information and educate people about depression and mood disorders, their causes and consequences. Second, to provide emotional and practical support to individuals and their families. Third, to support research into understanding and treating depression.

They achieve their objectives through the provision of over 2,000 face-to-face support groups nationwide that are facilitated by trained volunteers. They have recently started a online support group, beginning in 2011 with 5 users rising to 15 at the start of 2012. They have a low-cost telephone line that in 2011 took over 19,000 calls, again all manned by trained volunteers. Aware is a well recognized and respected brand name in Ireland and receive some government funding with 80% of their funding raised from public donations. Their user group is national and numbers of users high, therefore they are well placed to collaborate in providing a low-intensity depression treatment for the community that will include support for participants.

### Objectives of the trial

The study aims to implement and evaluate the efficacy of an online self-administered treatment for depression, with support, for users of the Aware Charity that have depressive symptoms. The research questions are:

▪ First, can an online treatment for symptoms of depression be effective as a low-intensity community-based intervention for adult users with depression?

▪ Second, what do participants find helpful and hindering in their online treatment for depression symptoms?

▪ Third, are participants satisfied with accessing and using an online treatment for depression symptoms?

▪ Fourth, what are the reasons for dropout from an online treatment?

Based on the success that has been achieved with supported online treatments for depression [34] we hypothesize that participants in the trial will demonstrate significant decreases in depressive symptoms post-intervention and a corresponding increase in quality of life indicators.

## Methods

### Study design

The study is a randomized controlled trial of an internet intervention for the treatment of depression. Participants will be randomized into two groups: the internet-delivered intervention with support and a waiting-list control group. The study protocol, information on the study, informed consent and related materials have been submitted and approved by the ethics committee at the School of Psychology, Trinity College Dublin (22/11/2013).

### Sample size

An analysis of a number of previous studies of an online intervention for depression in the community and that have used a CBT protocol similar to the intervention in the current study have reported post-treatment effect sizes averaging 1.29, and follow-up effects averaging 1.42, based on samples ranging from 45 – 300 [39,42-48]. We calculated power using G-Power software [49], using a power of 0.80 and an alpha of 0.05, we would need 60 subjects in each arm of the trial to observe a moderate (*d =* .50) post-treatment difference.

### Eligibility criteria

All adult users of Aware services will be eligible to participate. On screening participants, the following eligibility criteria will apply:

Exclusion criteria:

Suicidal intent/ideation: score >2 on BDI-II question 9

Psychotic illness

Currently in psychological treatment for depression

On medication for less than 1 month

Alcohol or drug misuse

Previous diagnosis of an organic mental health disorder

Depression preceding or coinciding a diagnosed medical condition

Inclusion Criteria

18 years of age

Mild to Moderate depressive symptoms (BDI-II 14–29)

### Recruitment

Aware is a charity that provides face-to-face, online and phone support for people experiencing depressive symptoms. The model is based on peer support but their trained volunteers also incorporate evidence-based life skills training for managing depression.

Potential participants will be able to visit a website to receive information on the study, what will be involved in participating, the treatment, and how to make contact and proceed with screening. On agreeing to participate, informed consent will be completed online and thereafter the baseline screening questionnaires.

### Randomization

**Figure 1:**
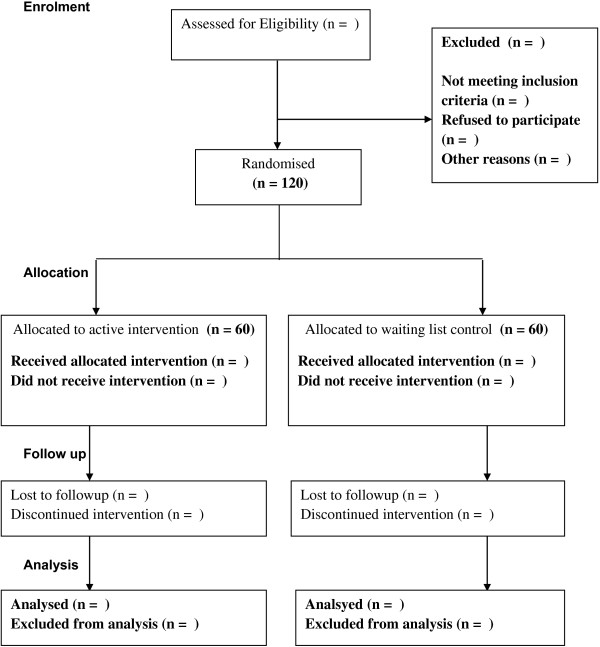
Flowchart of the study CONSORT.

Randomization will take place at the individual level after the baseline screening. Computer algorithms will determine scores on the screening questionnaires and will automatically assign participants to one of two groups – active intervention group or a waiting-list control group. Participants will be immediately informed about the randomization outcome. The randomization procedure will be managed by a person independent of the research group (Figure 1).

### Interventions

#### SilverCloud platform

Based on the inventive technologies that comprise the SilverCloud platform, the program for the treatment of depression employs several innovative engagement strategies for improving the user experience, described below:

1) *Personal* – The user has their own secure home page, which is about them and where they are in the program. For example, the user can fill in a profile with basic information about themselves. As well as establishing a sense of ownership, this information is also useful for the supporter, allowing them to provide more personal feedback. The homepage is intended to provide a reflective space; the user can document their thoughts and feelings, and these can be elaborated on within the journal application, which also acts as the vehicle for therapeutic writing exercises. The user has actions suggested to them, and as they complete modules of the program, their achievements are noted. Users are free to access the modules in any order they wish, in either a linear or non-linear manner, contributing to a sense of empowerment. As well as the central content being delivered, a range of satellite applications are provided, such as a goal-setting application, which can be used independently of the program content. Applications are released as the user completes modules, with the intention of maintaining engagement by introducing new features over time and not overwhelming the user initially. Users can also control which applications appear on their home page.

2) *Interactive* – The program includes a number of interactive elements and graphical exercises, which are aimed at engaging users with the therapeutic content, for example, reflecting on their own thinking. Users also have the ability to respond to content, indicating whether they like it, and also to comment on it. Both exercises and comments can be explicitly shared with the supporter. The user is provided with immediate feedback wherever possible; for example, when a charting exercise such as a mood chart is completed, the application item is graphically updated on the home page. Likewise, items are ticked off on the to-do list when completed, and achievements unlocked in each module summary.

3) *Supportive* – Each user has an assigned supporter, who provides weekly reviews of their progress on the program. This support is asynchronous, whereby the supporter sets a date to review their user’s progress, and they do not provide feedback, support or contact outside this time. The supporter can support multiple users, logging in once weekly for instance, and reviewing the work of all their online users within an allocated time period. Such asynchronous online contact may be logistically easier to implement for many services compared to motivational interviewing and telephone support. The system supports the exchange of messages between the user and supporter, but goes beyond electronic mail as the user is encouraged to share their content (such as completed exercises and comments) with their supporter. This shared content allows the supporter to respond in a more personal way and provide guidance as well as encouragement to keep using the program. Adherence information is also available to the supporter, and they can keep track of the user’s progress. This is all personally sensitive information, and so a shared view is provided in the user interface where they can see the supporter’s view of their data. By making the visibility of their data to the supporter more transparent, as well as the ability to explicitly change the sharing status of data, the user is provided with a greater sense of control while facilitating a meaningful interaction with the supporter.

4) *Social* – While group therapy and peer group support are well established, introducing contact with other users within any online system raises a number of ethical concerns regarding the possibility for unhelpful or negative content or communications. As a first step, the user can see anonymous indications of other people in the system. The intention is to reassure users that they are not alone in experiencing difficulties and that many other people have experienced similar problems and overcome them. Users can respond to content by indicating that they “like” it, and can see how many other people liked it, helping to reduce the sense of isolation. Other more detailed shared content (such as tips and ideas) is subject to supporter moderation.

#### Computerized cognitive-behavior therapy (iCBT) program

*Mind Balance* is a seven-module online CBT-based intervention for depression, delivered on a Web 2.0 platform using media-rich interactive content. The treatment comprises cognitive and behavioral components including self-monitoring and thought recording, behavioural activation, cognitive restructuring, and challenging core beliefs. The structure and content of the program modules follow evidence-based CBT principles. The content of each module is described briefly in Table 1 below. Each module is structured in an identical way and incorporates introductory quizzes, videos, informational content, interactive activities, as well as homework suggestions and summaries. In addition, personal stories and accounts from other users are incorporated into the presentation of the material.

**Table 1:** Mind Balance: description of module content

**Module name**	**Brief description**
Getting Started	Outlines the basic premise of CBT, some information about depression, and introduces some of the key ideas of *Mind Balance*. Users are encouraged to begin to chart their own current difficulties with depression.
Tune In I: Getting to Grips with Mood	The focus in this module is on mood monitoring and emotional literacy. Users can explore different aspects of emotions, physical reactions, action and inaction, and how they are related.
Tune in II: Spotting Thoughts	This module focuses on noting and tracking thoughts. Users can explore the connection between their cognitions and their mood, and record them graphically.
Change It I: Boosting Behaviour	This module focuses on behavioural change as a way to improve mood. Ideas about behavioural activation are included, and users can plan and record activities, and chart their relationship with their mood.
Change It II: Challenge Your Thoughts	This module supports users to challenge distorted or overly negative thinking patterns, with thought records, as well as helpful coping thoughts
Change It III: Core Beliefs	This module outlines the role that deeply-held core beliefs can play in mood and depression. Users can use a range of interactive activities to identify, challenge and balance any unhelpful core beliefs.
Bringing It All Together	In this final module, users are encouraged to bring together all the skills and ideas they have gathered so far, note their personal warning signs, and make a plan for staying well.

#### Waiting list control group

Participants in the waiting-list control group receive no treatment for the duration of the first 8 weeks. At week 8 waiting-list participants will complete the primary and secondary outcome measures (BDI-II, Sociodemographic & History, GAD-7, WSAS and EQ-5D) and will be given access to the program for 8 weeks with support from a trained volunteer.

### Support during treatment

Each participant will be assigned a supporter who will monitor their progress throughout the trial. Once a participant is assigned to the active treatment condition at their first login there will be a message from their supporter. This is a standard message addressed to the participant, welcoming them to the program, highlighting aspects of the program, encouraging them in the use of the program, and letting them know that each week their supporter will login and review their progress, leaving feedback for them and responding to the work they have completed. All supporters are presently trained supporters working with Aware. In addition, they will receive further training in the SliverCloud platform and how to deliver feedback (2 evenings 6–9 pm and one full day 10-5 pm).

Each supporter will be assigned 8 participants to provide post-session feedback of between 10–15 minutes per participant per session. As part of the online program, a dashboard interface will be provided to supporters that will give them an overview of their participant’s level of engagement with the program content. The supporters will respond to the work of their participants from session to session.

Supporters will form groups of five and have a senior supporter as a mentor. The senior supporters are volunteers with many years experience and have responsibility for inducting and training other volunteers within the Aware charity. Supporters will meet on a weekly basis for guidance on how support is delivered and discuss any difficulties. In addition, supporters will meet monthly with two senior trainers at Aware to review guidance and process case examples of feedback.

### Assessments

Participants will be assessed at baseline, posttreatment, and at 3 and 6 months follow-up. The study variables assessed are summerized in Table 2.

**Table 2:** Study measures to be used

**Measure**	**Assessment**	**Time of assessment**
Beck Depression Inventory-II (BDI-II)	Depression symptoms	Baseline, posttreatment and follow-up
Generalized Anxiety Disorder-7 (GAD-7)	Anxiety symptoms	Baseline, posttreatment and follow-up
Sociodemographic & History Questionnaire	Gender, age, marital status, education, occupation, socioeconomic status, and history	Baseline
Work and Social Adjustment Scale (WSAS)	Work and Social Adjustment scale	Baseline, posttreatment and follow-up
EuroQol 5D (EQ-5D)	Quality of Life	Baseline, Posttreatment and follow-up
Engagement and Usage data	Engagement and usage	Continuous
Satisfaction with Treatment (SAT)	Satisfaction with therapy	Posttreatment
Helpful and Hindering Aspects of Treatment (HAT)	Helpful and hindering aspects of therapy	After each session

At baseline, assessments include the Beck Depression Inventory-II (BDI-II), Sociodemographic & History Questionnaire, Generalized Anxiety Disorder-7 (GAD-7), and the Work and Social Adjustment Scale (WSAS), and EuroQol 5D (EQ-5D) will be completed for screening purposes. Thereafter the BDI-II, GAD-7, EQ-5D and WSAS will be completed at the end of treatment, week 8, and at follow-up, week 20 (3-months) and week 32 (6-months). After each session participants will be asked to complete the Helpful Aspects of Treatment Form (HAT). The measure Satisfaction with Treatment (SAT) will be administered at week 8.

### Measures

#### Screening measure

***Sociodemographic Information & History Questionnaire*** will be developed based on a previous version [39] and will collect demographic details on the participants. It collects data on previous diagnosis of anxiety disorders and length of time that one is experiencing depression symptoms. It will collect data on participant’s experience of counseling/therapy and medication for depression. Data is collected on whether one has a previous diagnosis of an organic mental health disorder such as schizophrenia, psychosis, bipolar disorder. In addition, it contains items related to co-morbidity of depression with presence of psychosis, alcohol and drug misuse, and/or any recent medical diagnosis.

#### Primary outcome measure

***The Beck Depression Inventory*** (BDI-II) [50] The 21-item Beck Depression Inventory – Second Edition (BDI-II) is a widely used questionnaire developed for the assessment of depressive symptoms that correspond to the criteria for depressive disorder diagnosis as outlined in The American Psychiatric Associations Diagnostic and Statistical Manual of Mental Disorders–Fourth Edition (DSM-IV) [51]. Each item is scored on a scale from 0 to 3. The BDI-II manual states that a cut score of 17 has yielded a 93% specificity and 18% sensitivity for the presence of major depression (Beck et al., [50]). The scale designates levels of severity, Minimal (0–13); Mild (14–19); Moderate (20–28); and Severe (29–63) [50].

The BDI-II has been found to have excellent internal consistency and test–retest reliability with a diverse range of samples [50,52,53]. The BDI-II has demonstrated good convergent validity with other measures of depression among clinical and nonclinical adult samples [54].

#### Secondary outcomes

***General Anxiety Disorder (GAD-7)*** [55] comprises 7 items measuring symptoms and severity of GAD based on the DSM-IV diagnostic criteria for GAD. The GAD-7 has good internal consistency (.89) and good convergent validity with other anxiety scales [56]. Increasing scores indicating greater severity of symptoms [57]. The GAD-7 is increasingly used in large-scale studies as a generic measure of changes in anxiety symptomatology, using a cut-off score of 8 [31,58].

**Work and Social Adjustment (WSAS)**[59] The WSAS is a simple, reliable and valid measure of impaired functioning. It is a simple 5-item self-report measure which provides an experiential impact of a disorder from the patient’s point of view. It looks at how the disorder impairs the patient’s ability to function day to day on five dimensions: work, social life, home life, private life and close relationships.

**EuroQol 5D (EQ-5D)**[60]. Is a generic instrument of health-related quality of life: Part 1 records self-reported problems in each of five domains: mobility, self-care, usual activities, pain/discomfort and anxiety/depression. Each domain is divided into three levels of severity corresponding to no problems, some problems, and extreme problems, which allows obtaining a population-based preference score or societal index (SI). Part 2 records the subject’s self-assessed health on a Visual Analogue Scale (VAS), a 10 cm vertical line on which the best and worst imaginable health states score 100 and 0, respectively.

### Other measures

***Helpful Aspects of Therapy Form (HAT)*** [61,62] is an instrument that assesses the most helpful and hindering events in the therapy. The user is asked to describe in their own words whether there was anything particularly helpful or hindering in the session. Participants are asked to describe any event, anything they engaged with in the session that was helpful or hindering for them.

***Satisfaction with Treatment (SAT)*** [39] At post-treatment participants will be asked to complete a satisfaction with treatment measure. The measure is based on a Net Promotoer Score: which is a management tool that can be used to gauge the loyalty of a firm’s customer relationships. It serves as an alternative to traditional customer satisfaction; it includes one question: How likely is it that you would recommend this treatment to a friend or colleague? Descriptive statistics will be used to report on the data from the other quantitative questions on the SAT measure. The satisfaction measure will also contain two questions asking to describe what they most liked and least liked about the online treatment.

#### Pre-treatment and during treatment dropout questionnaires

Two simple questions will be developed for the study, one asking about the reasons for deciding to dropout without beginning treatment and the second asking about the reasons for dropping out during treatment. The link for each of these will be contained in the appropriate emails that clinicians send to their respective participants, following the protocol: Pre-treatment dropout - after **one** week the clinician can send the questionnaire by e-mail. Participants discontinue treatment, after **one** missed sessions the clinican should send a reminder message to the participant by email. If after **one** further week the passrticipant has not responded the clinician can send the questionnaire asking about the reasons for dropout.

### Engagement and usage measures

The online system will collect anonymized descriptive information relating to engagement and usage. Data collected will include the number of sessions completed; mean time spent on the program, average number of sessions per user, and average length of a session. A session is defined as an instance where a user logs on to the system. Session time estimation will always be an imperfect calculation, as users may be interrupted or take breaks within a session, and may not formally log out of the system. All user activity within the system such as reading a content page, saving a journal entry, or updating an activity, is logged with a time stamp. Starting with the log entry of the user logging on, the total time is calculated by adding up the time that elapses between each subsequent log record (in the same manner as popular web analytics software). On its own, this will yield a result vulnerable to overestimation of session time. To avoid counting periods where the user is not actively engaged with the system, any interactions taking longer than 30 minutes are counted as 1 minute. Any period of inactivity longer than 3 hours will start the count on a new session, rather than extending the time of the current session. Use of different program components will be measured. Data related to supporter reviews will be collected.

### Ethical considerations

Informed consent will be obtained from each participant before randomization. Before consent participants will be made fully aware of what is involved in participating and especially the importance of the waiting-list control group. They will be familiar with the aims and objectives of the study and why the study is important. They will also be informed that they will be participating voluntarily and if they so wish can withdraw at any time. At baseline if participants are excluded on the basis of the established exclusion criteria they will be referred to other appropriate sources of support such as Aware Charity telephone line and e-mail support.

### Planned statistical analysis

The analyses will be based on the intention-to-treat principle, including those who began treatment and provided follow-up data irrespective of treatment compliance. Effects will be tested at the 0.05 level. All analyses will be carried out using SPSS. Baseline differences in demographic and clinical characteristics will be investigated using ANOVA. To assess for significant changes over time, repeated measures ANOVAs will be performed for the primary outcome measure for depression (BDI-II). In case of significant time x group interactions, contrasts will be conducted comparing changes from baseline to post-treatment for each group separately. Further any relationships between baseline sociodemographic data and outcomes on the primary outcome measure will be assessed. Repeated measures ANOVAs will also be used to assess any changes over time for secondary outcomes (GAD-7, EQ-5D and WSAS).

The magnitude of the within-group effects of each of the interventions (Cohen’s *d*) will be calculated using the pooled standard deviation. The magnitude of between-group effects will be established by subtracting the Cohen’s *d* statistic for the two groups [63]. For Cohen’s *d* an effect size of 0.2 to 0.3 can be considered a small effect, around 0.5 a medium effect and 0.8 upwards, a large effect [64].

Analysis will be made to determine the proportion of participants who make clinically meaningful change at end of treatment and at follow-up. An assessment of *remission* will be made using pre-treatment compared to post-treatment and follow-up scores on the primary outcome measure (BDI-II) and also for the secondary outcomes (GAD-7). The assessment of *remission* will consider the following cut-off scores for the primary outcome measure BDI-II score of <14 [50], and GAD-7 total score ≤8 [57] and and WSAS <10 [59]. In addition, using the post-treatment scores for depression (BDI-II) and anxiety (GAD-7) and dividing these with their pre-treatment scores a calculation of relative risk of depression and anxiety can be made [58].

An index of *recovery* will be established by identifying the percentage of participants whom achieved a 50% reduction in pre-treatment compared to post-treatment score on BDI-II and GAD-7 [58]. In addition we will also calculate the number of reliably changed and recovered participants using Jacobson and Truax’s [65] criteria.

To analyse the HAT data we will use the descriptive and interpretative framework described by Elliott and Timulak [66]. Participants’ responses will be considered within domains of helpful and non-helpful events and impacts. Firstly, individual units of text that could stand meaningfully out of their context will be identified. Next each of these will be organized into domains of helpful events and helpful impacts. Similar events and impacts will be grouped into categories, which will be then finalized and suitably named and defined. The process is organic, involving constant reference to the source data [67].

Descriptive statistics will report on the results from the quantitative questions on satisfaction. The SAT data collected from the two open questions will be analysed qualitatively following the descriptive and interpretative framework described by Elliott and Timulak [64,68]. Similarly, data from droput pre-treatment and during treatment will be analysed qualitatively.

## Discussion

The study seeks to evaluate the efficacy and effectiveness of an online delivered treatment for depressive symptoms in a sample of adult users of a charity service. The study will be a first contribution to the potential for An online low-intensity intervention to demonstrate decreases in depressive symptoms for participants as compared to the waiting-list control. The study will be a contribution to the already established work in online treatments for depression worldwide.

The primary outcome measure (BDI-II) that will assess depressive symptoms is a well established measure and has been used in other trials of internet-delivered treatments. The secondary outcome measures (GAD-7, HAT, SAT, EQ-5D, WSAS) included in the study will contribute to what participants find satisfying with online treatments and further will detail what in-session events and their impacts participants report as being helpful or hindering in their online treatments. The WSAS will yield information on improvements in quality of life for participants, as will the EQ-5D. The GAD-7 will shed light on the prevalence of comorbid anxiety disorder that occurs with depression in the sample.

It is hoped that with the inclusion of pre-treatment and during treatment dropout questionnaires that the study can make a contribution to developing a better understanding of the reasons for dropout in online treatments.

Depending on the sample recruited the results may be generalizable to people with mild to moderate symptoms of depression in Ireland and may therefore give insight into the potential of low-intensity interventions for Irish people with depressive symptoms. The internet-based intervention is probably most interesting for people who look for alternative healthcare because the possibility of accessing traditional services is near impossible, prohibitive due to waiting lists, costs, and personal stigma.

While the study will not include an official diagnosis of participants, it does include established measures that will gather valuable data on participants that can allow us to begin to build a picture of the relevance of iCBT treatments for depression in Ireland.

### Trial status

Currently we are preparing all necessary materials for the launch of the trial in January 2014.

## Competing interests

GD, JS, declare a minority interest in the commercialization of the SilverCloud platform.

## Authors’ contribution

DR, LT, GD & JS are principal investigators for the project. DR, LT, GD, JS conceptualized the initial trial design. This was developed with the help of the team members. The manuscript was written by DR, with significant contributions and revisions from LT, GD. DR distributed the manuscript to the group for discussion and revisions. The finalized manuscript was agreed by all authors.

## Pre-publication history

The pre-publication history for this paper can be accessed here:

http://www.biomedcentral.com/1471-244X/14/147/prepub
